# Retrospective Cohort Analysis of the Effect of Antimicrobial Stewardship on Postoperative Antibiotic Therapy in Complicated Intra-Abdominal Infections: Short-Course Therapy Does Not Compromise Patients’ Safety

**DOI:** 10.3390/antibiotics11010120

**Published:** 2022-01-17

**Authors:** Güzin Surat, Pascal Meyer-Sautter, Jan Rüsch, Johannes Braun-Feldweg, Christoph-Thomas Germer, Johan Friso Lock

**Affiliations:** 1 Unit for Infection Control and Antimicrobial Stewardship, University Hospital of Würzburg, 97080 Würzburg, Germany; 2 Department of General-, Visceral-, Transplant-, Vascular- and Pediatric Surgery, University Hospital of Würzburg, 97080 Würzburg, Germany; meyer-sautter.p@gmx.de (P.M.-S.); jan-ruesch@t-online.de (J.R.); johannes.braun-feldweg@gmx.de (J.B.-F.); germer_c@ukw.de (C.-T.G.); lock_j@ukw.de (J.F.L.)

**Keywords:** antimicrobial stewardship, antibiotic prescribing quality, low-risk intra-abdominal infections, post-operative antibiotic treatment

## Abstract

Background: Recent evidence suggests that short-course postoperative antibiotic therapy (PAT) of intra-abdominal infections is non-inferior considering clinical outcomes. The aim of this study was to compare the outcome of short vs. long PAT in complicated intra-abdominal infections (cIAIs) without sepsis. Methods: We performed a single center-quality improvement study at a 1500 bed sized university hospital in Bavaria, Germany, with evaluation of the length of antibiotic therapy after emergency surgery on cIAIs with adequate source control during 2016 to 2018. We reviewed a total of 260 cases (160 short duration vs. 100 long duration). The antibiotic prescribing quality was assessed by our in-house antimicrobial stewardship team (AMS). Results: No significant differences of patient characteristics were observed between short and long PAT. The frequency of long PAT declined during the observation period from 48.1% to 26.3%. Prolongation of PAT was not linked with any clinical benefits, on the contrary clinical outcome of patients receiving longer regimes were associated with higher postoperative morbidity. AMS identified additional educational targets to improve antibiotic prescribing quality on general wards like unnecessary postoperative switches of antibiotic regimes, e.g., unrequired switches to oral antibiotics as well as prolongation of PAT due to elevated CRP. Conclusion: Short-course antibiotic therapy after successful surgical source control in cIAIs is safe, and long-duration PAT has no beneficial effects.

## 1. Introduction

Antimicrobial stewardship programs (ASPs) are gaining, globally, increasing in merited recognition and acceptance and were primarily launched to stop antimicrobial resistance (AMR) [1,2]. As antibiotic consumption is considered the main driver for AMR—natural factors, such as intrinsic or acquired genetic resistance patterns, environmental sources and missing hygiene measures are contributing effects too—one of the starting-points includes improving the social and prescribing attitude towards the use of antimicrobial agents [3,4,5,6]. Indication, choice of antimicrobial agent, way of application, de-escalation efforts and duration are amongst the markers to be evaluated each time antibiotics are prescribed [7,8,9]. Incorporating multimodal concepts by engaging the responsible physicians without neglecting nurse staff and undergraduate trainees embedded in a multidisciplinary team, including infectious diseases specialists, microbiologists, pharmacists and infection control physicians in charge, is by far the most worthwhile strategy in order to assure sustainable success for AMR to be antagonized [10,11,12,13]. Postantibiotic duration for complicated surgical intra-abdominal infections (cIAIs) attracts focus and motivates progressively more data suggesting that a short regimen may suffice for an optimal clinical recovery applying for both, complicated mild/moderate IAIs and severe postoperative IAIs in critically ill patients, provided source control has been achieved [14,15,16]. Maximizing the clinical benefit by minimizing collateral damage remains the curative principle especially given the high rates of mortality and morbidity in patients with (uncontrolled) cIAIs [17,18,19]. Uncomplicated IAIs are managed either only surgically or conservatively with antibiotics alone. For cIAIs the approach encloses timely performed surgical source control with appropriate antimicrobial treatment; community or hospital acquired IAIs may be uncomplicated or complicated by definition as well [17,20,21,22].

This study is to be understood as a sequel to previously published data by Surat et al. on the impact of antimicrobial stewardship on antibiotic consumption for non-elective surgical IAIs [12]. However, this sub-analysis opens the chapter to postantibiotic therapy (PAT) in complicated mild/moderate community-acquired IAIs in non-septic patients with achieved source control, also encompassing de-escalation manners e.g., switching to oral therapy conducted on general wards and aim at confirming the trend that a short duration of PAT is again not afflicted with higher rates of postoperative infectious complications or worse clinical outcomes.

## 2. Methods

This quality improvement study entails a period of 3 years (2016–2018) and was conducted retrospectively in a 1500 bed tertiary hospital in Germany, with an in-hospital ASP officially launched in 2015, gradually reaching out to all departments including the department of general surgery by 2018. The backbone of the in-house AMS team consists of infection control physicians, microbiologists, pharmacists and infectious diseases (ID) consultants with an ID physician responsible for the leadership. The prequel of this project included 776 patients and focused on the impact of antimicrobial stewardship interventions on surgical antibiotic prescription behavior of surgical IAIs, especially postoperative antibiotic use and the appropriateness of indication. The previous analysis revealed a significant reduction of total days of antibiotic therapy and fewer patients receiving PAT altogether [12]. The intention of this subsequent analysis was to assess the impact of antimicrobial stewardship implementations on patients actually receiving PAT due to cIAIs but were non-septic or had life-threatening conditions.

### 2.1. Study Design

The effects of different durations of antibiotic therapy in IAIs were examined by a retrospective cohort analysis. All data were retrieved from the hospital information system and transferred in a pseudonymous database with multiple variables containing baseline patient characteristics, pre-, peri- and postoperative antibiotic therapy (ABT), surgical therapy, and postoperative 30-day outcome. We defined two groups based of the duration of PAT. The short duration group was limited to a maximum of 4 days post-surgery, leant on the STOP-IT trial by Sawyer et al. (sPAT group) [14]. Patients with longer PAT were included in the lPAT group. Any extension beyond this had to be discussed with the in-hospital AMS-team. Reasons for allowed extensions of therapy were immune suppression or other present infections such as pneumonia or urinary tract infection. The follow-up was limited to 30 days.

### 2.2. Patients

All patients ≥18 years undergoing emergency abdominal surgery with IAI and PAT during 01.01.2016 and 31.12.2018 were included with the following selection criteria: Diagnosis of peritonitis (ICD-10 K65.0–K65.9), acute cholecystitis (ICD-10 K80.0-K80.01, K81.0), acute appendicitis (ICD-10 K35.2–K35.8), acute diverticulitis (ICD-10 K57.2–K57.22), or intestinal perforation (K25.1–K25.2, K26.1–K26.2, K63.0–K63.2). Patients with the following criteria were excluded from analysis: Acute pancreatitis, acute mesenteric ischemia, acute leukemia, end-stage malignant disease in palliative care, ASA score > IV, extra-abdominal infectious focus requiring antimicrobial therapy before and after surgery. For this subgroup analysis, we included only non-septic patients with complicated IAIs with successfully achieved surgical source control. Patients with postoperative anastomotic insufficiency were excluded in this analysis.

### 2.3. Outcome Assessment

Postoperative outcome assessment up to 30 days postoperative. Postoperative complications were graded according to Clavien-Dindo [23]. Clavien-Dindo grade I-II complications were appraised as no severe complications, whereas Clavien-Dindo grade IIIa-V complications were appraised as severe complications. Surgical site infections (SSI) were defined according to the Centers for Disease Control and Prevention (CDC) criteria [24].

### 2.4. Statistical Analysis

All statistical analyses were performed using IBM SPSS Statistics, version 26 (International Business Machines Corporation, Armonk, NY, USA). Descriptive data were reported as means with standard deviation, unless otherwise noted. Groups were compared using the Chi-square, Fisher’s exact Test or Mann–Whitney U test according to the data scale and distribution. The level of statistical significance was 0.05 (two-sided).

## 3. Results

### Patients’ Baseline Characteristics and Indications for Emergency Surgery

There were no significant disparities in the preoperative risk-stratification between the two groups. Shorter therapies were significantly more common in 2018 than in the previous two years. The collected risk scores (Charlson comorbidity index and ASA score) did not either differ significantly between both groups. Severe previous liver or kidney disease or immunosuppression at the time of surgery were generally rare in the observed cohort and similarly distributed between both groups. Preoperative risk factors such as prolonged hospitalizations or pre-operations were also not present in greater numbers in either group. Intraoperative findings revealed a higher prevalence of peritonitis in the sPAT group (Table 1). There were slightly more cases of cholecystitis in the sPAT, and slightly more cases of appendicitis and colonic perforations in the lPAT group.

Insignificantly more patients (sPAT 50% vs. lPAT 38%) were admitted directly to the normal ward compared to patients who required intensive care support (sPAT 33.8% vs. lPAT 48%). Accordingly, these patients were more often postoperatively ventilated (sPAT 21.3% vs. lPAT 31%) and received vasopressors (sPAT 17.5% vs. lPAT 26%). However, these differences were again not statistically significant. There were almost twice as many surgical side infections in the lPAT group (sPAT 6.9% vs. lPAT 12%), almost as many as non-intra-abdominal infections (lPAT 11.9% vs. lPAT 10%), but this effect was also not statistically significant. Importantly the groups differed significantly regarding postoperative complications. The rate of necessary re-interventions was almost twice as high in long-treated patients (sPAT 15% vs. lPAT 27%). Of these re-interventions many had to be performed as re-operations (sPAT 8.8% vs. lPAT 23%). Accordingly, the postoperative complications classified as per Clavien–Dindo were found to be to the disadvantage of the lPAT group (sPAT 11.9% vs. lPAT 23%) (a complication-free course was significantly more frequent in the short treated group (sPAT 36.3% vs. lPAT 16%). Length-of-stay (LOS) differed significantly in the sPAT group (median 7 days) compared to the lPAT group (median 11 days). In contrast, there was no difference in LOIS (Table 2). While the total duration of PAT in the short-treated group was 4 days on average and median, patients in the lPAT group were treated for more than twice as long (median 8; Table 2).

The initial empiric antibiotic regimens between both groups were quite similar. Patients on long therapy were more frequently subject to switches (sPAT 19.4% vs. lPAT 56%). Switches were rarely due to AMS recommendations in either group (sPAT 9.7% vs. lPAT 1.8%), nor to keeping with the actual resistograms. Undocumented indications for the use of antibiotics were still high (sPAT 77.4% vs. lPAT 72.7%; Table 3). Nevertheless, most indications were deemed appropriate by our in-house AMS-team (sPAT 75.6% vs. lPAT 77%). Inappropriate indications were mostly due to prolongations of the perioperative prophylaxis (PAP). There were large differences in the management of switches, for example 32% of those treated long were incorrectly escalated (mostly from a 1st/2nd generation cephalosporin to an oral 3rd generation cephalosporin, in comparison to 9.4% in the short-treated group (Table 3). As per general definition a switch form intravenous to oral antibiotic therapy is considered de-escalation, we defined this step as ‘escalation’ when the selected oral antibiotic belonged to 3rd generation cephalosporins such as cefpodoxime [7].

## 4. Discussion

In this retrospective single-center study we analyzed patients requiring emergency surgery for complicated IAIs over 2016–2018 with attention on the length of PAT. Yet, unlike to the prequel published by Surat et al. these findings included only non-septic patients with adequate source control [12,25]. This time the prescribing attitudes of surgeons on general wards were the focus of our observations, within the wider ambition of discerning the influence of biochemical inflammation markers such as C-reactive protein (CRP) or procalcitonin (PCT) on the duration of PAT.

In accordance with the data released on postsurgical antimicrobial management in complicated community acquired (or healthcare associated IAIs) so far, our results support that shortened PAT is not associated with worse clinical outcomes. The surgical and clinical conditions that warranted interventions in this study were similar to the general published data (e.g., peritonitis, appendicitis, cholecystitis) [14,15,26,27,28]. Here, both groups did not differ in the risk-profile and yet the long-duration arm became evident with a significantly higher rate of infectious complications and, in consequence necessitated more re-operations. Continuing misuse of antibiotics has been linked with avoidable adverse events, emergence of antibiotic resistance and unnecessary monetary burden for the health system and demands a change in the prescribing culture of antibiotics [9,29]. The debate about the duration of PAT is still ongoing and remains an important key factor for ASPs to target on for it is deemed to being the main reason for inappropriate use of antibiotics in managing IAIs [20,30]. Fortunately, our results re-emphasize the role of ASPs on antibiotics for the postsurgical therapy of complicated IAIs: over the observed three years (2016–2018) the duration of PAT successively shortened, which is mainly attributed to the roll-out of our in-hospital ASP involving general wards in the already regularly happening antibiotic ward rounds and discussions in intensive care units. Although this finding was not statistically significant, long-duration PAT, on the other hand, did not prevent the significant need for re-interventions, even given the fact that PAT was administered twice as long within the long-duration group. These results are in line with data from Tellado et al. that showed that inappropriate indication for the empiric use of antibiotics was associated with unsuccessful outcomes and a higher rate of e.g., re-operations [31].

Looking further into the quality of antibiotic utilization, the long-duration group happened to have not only a higher rate of switches of the empirically selected antibiotic agents, but these switches mainly took place on the general surgical wards resulting in ‘escalations’ to oral antibiotics–reasonable, one might think given patients surgical and clinical status and the fact that AMS consultations for general wards were missing at the time. Although intravenous to oral antibiotic switch is a main tool in ASPs, in our study these actions were considered inappropriate by our in-hospital AMS team for it prolonged unnecessarily the duration wherein treatment could have been stopped. Importantly, the choices of oral antibiotics were not in keeping with the in-house AMS de-escalation standards (2nd- and 3rd-generation cephalosporin with poor oral bioavailability lacking efficacy; data not available in the result part) [7,9,28].

Guiding antibiotic therapy by inflammatory markers (e.g., leukocyte count, CRP, PCT or interleukin 6), has been numerously investigated in hospitalized patients including those critically ill. PCT carries more specificity and sensitivity in the detection of truly bacterial infections and the guidance of antibiotic duration by PCT may result in significant reduction of antibiotic consumption and mortality [32,33,34,35,36]. Data on the prescribing behavior in cIAIs directed by named markers remain sparse and yet the results, so far, attest PCT a useful tool for both the diagnosis of bacterial infections and discontinuation of antibiotics; yet, it must be stressed that biomarkers should not be read outside the clinical setting [35,37,38]. The power and the nature of this study does not allow to draw a conclusion regarding the role of PCT in terms of ceasing PAT or the safeness of such a course, yet following the decrease of the CRP level was associated with longer PAT.

Discussing the results of this study on the whole, the point of its research nature as in the meaning of monocentric and retrospective limits their interpretation. Furthermore, the power of the study cannot be used to reason that shortened duration of PAT is associated with improved outcomes, but it clearly suggests that a longer duration of antibiotic therapy is tied with more complications and does do more harm than good. The subject of the influence of laboratory markers on the duration of PAT and the detailed appropriateness and quantification of switches from intravenous to oral antibiotics will be outlined in future investigations. In conclusion, our results confirm that short-course antibiotic therapy after successful surgical source control in cIAIs is safe.

## Figures and Tables

**Table 1:** Preoperative patient characteristics and intraoperative findings.

Characteristic	Patients, No. (%)		*p* Value ^b^
	Postoperative Antibiotic Therapy		
	Short (*n* = 160)	Long (*n* = 100)	
2016	42 (51.9)	39 (48.1)	0.015
2017	59 (59.6)	40 (40.4)	
2018	59 (73.8)	21 (26.3)	
age, mean (median)	58.00 (61.50)	58.40 (62.00)	0.910
ASA classification			
1	15 (9.4)	8 (8.0)	0.281
2	77 (48.1)	43 (43.0)	
3	58 (36.3)	36 (36.0)	
4	9 (5.6)	13 (13.0)	
BMI, mean (median)	27.30 (27.00)	27.00 (27.0)	0.832
CCI			
none (0)	41 (25.6)	27 (27.0)	0.264
low (1–2)	33 (20.6)	17 (17.0)	
moderate (3–4)	52 (32.5)	25 (25.0)	
severe (>4)	34 (21.3)	31 (31.0)	
liver cirrhosis	1 (0.6)	1 (1.0)	0.736
chronic kidney disease	15 (9.4)	17 (17.0)	0.069
current immunosuppressive drugs	9 (5.6)	8 (8.0)	0.451
community-acquired IAI	133 (83.1)	83 (83.0)	0.979
hospital-aquired IAI	27 (16.9)	17 (17.0)	
high-risk of MDR	28 (17.5)	17 (17.0)	0.917
preoperative ^a^ LOS, mean (median), d	14.00 (0.00)	13.00 (0.00)	0.724
surgery	15 (9.4)	8 (8.0)	0.704
MDR	5 (3.1)	5 (5.0)	0.444
MRSA	1 (0.6)	0 (0.0)	0.737
VRE	2 (1.3)	2 (2.0)	
3MRGN	1 (0.6)	2 (2.0)	
intraoperative peritonitis	90 (56.3)	49 (49.0)	0.254
gastric perforation	10 (6.3)	4 (4.0)	0.612
small intestine perforation	10 (6.3)	9 (9.0)	
colonic perforation	20 (12.5)	17 (17.0)	
appendicitis	55 (34.4)	39 (39.0)	
cholecystitis	57 (35.6)	28 (28.0)	
intestinal obstruction	7 (4.4)	3 (3.0)	

^a^ Within 30 days prior index surgery; ^b^ *p* values were derived from Chi-square, Fisher’s exact or Mann-Whitney U tests, depeding upon data scale. Abbreviations: ASA, American Society of Anesthesiologists; BMI, body mass index; CCI, Charlson comorbidity index; IAI, intra-abdominal infection; LOS, length of hospital stay; ABT, antibiotic therapy; MDR, multidrug-resistant bacteria, GI: gastrointestinal.

**Table 2:** Postoperative outcome.

Characteristic ^a^	Patients, No. (%)		*p* Value ^d^
	Postoperative Antibiotic Therapy		
	Short (*n* = 160)	Long (*n* = 100)	
postoperative transfer to			
general ward	80 (50.0)	38 (38.0)	0.069
IMC	26 (16.3)	14 (14.0)	
ICU	54 (33.8)	48 (48.0)	
postoperative organ support			
ventilation	34 (21.3)	31 (31.0)	0.077
vasopressors	28 (17.5)	26 (26.0)	0.100
SSI	11 (6.9)	12 (12.0)	0.157
other postoperative infections ^b^	19 (11.9)	10 (10.0)	0.640
re-intervention necessary	24 (15.0)	27 (27.0)	0.018
re-operation necessary	14 (8.8)	23 (23.0)	0.001
postoperative findings			
MDR	4 (2.5)	3 (3.0)	0.809
postoperative complications ^c^			
none	58 (36.3)	16 (16.0)	0.001
no severe complications	83 (51.9)	61 (61.0)	
severe complications	19 (11.9)	23 (23.0)	
postoperative mortality	2 (1.3)	0 (0)	0.262
LOS mean (median)	10.00 (7.00)	14.00 (11.00)	<0.001
LOIS mean (median)	2.00 (1.00)	3.00 (1.00)	0.138
duration of PAT mean (median) in days	4 (4)	9 (8.5)	<0.001

^a^ Within 30 days after the index surgery; ^b^ non-intraabdominal infection such as urinary tract infection, pneumonia, etc; ^c^ according to the Clavien–Dindo classification; ^d^ *p* values were derived from Chi-square, Fisher’s exact or Mann-Whitney U tests, depeding upon data scale. Abbreviations: IMC, intermediate care unit; ICU, intensive care unit; SSI, surgical site infection; MDR, multi-drug-resistance bacteria discovered postoperative; PAT, postoperative antibiotic therapy; AMS, antimicrobial stewardship; LOS, length of stay; LOIS, length of stay on ICU.

**Table 3:** Postoperative antibiotic therapy.

Characteristic	Patients, No. (%)		*p* Value ^a^
	Postoperative Antibiotic Therapy		
	Short (*n* = 160)	Long (*n* = 100)	
Initial Regimen:			
cephalosporins	76 (72.4)	52 (67.5)	0.641
broad-spectrum penicillin	26 (24.8)	21 (27.3)	
carbapenems	3 (2.9)	4 (5.2)	
switch of antibiotic agent	31 (19.4)	56 (56.0)	<0.001
postoperative day of switch, mean (median), d	3.00 (2.00)	4.00 (3.00)	0.004
Reason for Switch of Antibiotic Agent			
not documented	24 (77.4)	40 (72.7)	0.123
resistogram	4 (12.9)	14 (25.5)	
AMS council	3 (9.7)	1 (1.8)	
switch in ICU or IMC	7 (22.6)	9 (16.4)	0.567
switch on general ward	24 (77.4)	46 (83.6)	
Assessment Based on AMS-Guidelines			
PAT necessary	121 (75.6)	77 (77.0)	0.800
de-escalation or discontinuation correct	154 (96.3)	79 (79.0)	<0.001
missing de-escalation	4 (2.5)	20 (20.0)	
missing escalation	2 (1.3)	1 (1.0)	
Switch of Empirical Antibiotic Therapy			
not required or correctly performed	143 (89.4)	65 (65.0)	<0.001
wrong de-escalation	2 (1.3)	3 (3.0)	
wrong escalation	15 (9.4)	32 (32.0)	
efficacy			
not effective against strains	96 (60.0)	57 (57.0)	0.632
effective against detected strains	64 (40.0)	43 (43.0)	
Biochemical Values After PAT			
leukocytes. mean (median)	9.60 (8.60)	10.20 (9.90)	0.076
CRP mean (median)	10.30 (8.00)	6.10 (4.00)	<0.001
PCT mean (median)	6.90 (0.80)	0.50 (0.50)	0.643

^a^*p* values were derived from Chi-square, Fisher’s exact or Mann-Whitney U tests, depeding upon data scale. Abbreviations: AMS, antimicrobial-stewardship as defined by current AMS-standards; ICU, intensive care unit; IMC, intermediate care unit; PAT, postoperative antibiotic therapy; CRP, C-reactive protein; PCT, procalcitonin.

## Data Availability

The data presented in this study are available on request from the corresponding author. The data are not publicly available due to European General Data Protection Regulation (GDPR).
